# Prevalence and Causes of Blindness and Low Vision in Southern Sudan 

**DOI:** 10.1371/journal.pmed.0030477

**Published:** 2006-12-19

**Authors:** Jeremiah Ngondi, Francis Ole-Sempele, Alice Onsarigo, Ibrahim Matende, Samson Baba, Mark Reacher, Fiona Matthews, Carol Brayne, Paul M Emerson

**Affiliations:** 1 Department of Public Health and Primary Care, Institute of Public Health, University of Cambridge, Cambridge, United Kingdom; 2 Christian Mission Aid, Nairobi, Kenya; 3 The Carter Center, Nairobi, Kenya; 4 Ministry of Health, Government of South Sudan, Juba, South Sudan; 5 Medical Research Council Biostatistics Unit, Institute of Public Health, Cambridge, United Kingdom; 6 The Carter Center, Atlanta, Georgia, United States of America; Bradford Royal Infirmary, United Kingdom

## Abstract

**Background:**

Blindness and low vision are thought to be common in southern Sudan. However, the magnitude and geographical distribution are largely unknown. We aimed to estimate the prevalence of blindness and low vision, identify the main causes of blindness and low vision, and estimate targets for blindness prevention programs in Mankien payam (district), southern Sudan.

**Methods and Findings:**

A cross-sectional survey of the population aged 5 y and above was conducted in May 2005 using a two-stage cluster random sampling with probability proportional to size. The Snellen E chart was used to test visual acuity, and participants also underwent basic eye examination. Vision status was defined using World Health Organization categories of visual impairment based on presenting visual acuity (VA). A total of 2,954 persons were enumerated and 2,499 (84.6%) examined. Prevalence of blindness (presenting VA of less than 3/60 in the better eye) was 4.1% (95% confidence interval [CI], 3.4–4.8); prevalence of low vision (presenting VA of at least 3/60 but less than 18/60 in the better eye) was 7.7% (95% CI, 6.7–8.7); whereas prevalence of monocular visual impairment (presenting VA of at least 18/60 in better eye and VA of less than 18/60 in other eye) was 4.4% (95% CI, 3.6–5.3). The main causes of blindness were considered to be cataract (41.2%) and trachoma (35.3%), whereas low vision was mainly caused by trachoma (58.1%) and cataract (29.3%). It is estimated that in Mankien payam 1,154 persons aged 5 y and above (lower and upper bounds = 782–1,799) are blind, and 2,291 persons (lower and upper bounds = 1,820–2,898) have low vision.

**Conclusions:**

Blindness is a serious public health problem in Mankien, and there is urgent need to implement comprehensive blindness prevention programs. Further surveys are essential to confirm these tragic findings and estimate prevalence of blindness and low vision in the entire region of southern Sudan in order to facilitate planning of VISION 2020 objectives.

## Abstract 2

A cross-sectional survey using two-stage cluster random sampling was conducted in Mankien district, southern Sudan. The prevalence of blindness (4.1%) and of low vision (7.7%) were much higher than expected.

## Editors' Summary

**Background.:**

Blindness is very common. The World Health Organization says that around 161 million people have at least some degree of “visual impairment,” of whom 37 million are blind. There are many causes of blindness, including infections, malnutrition, injury, and aging. Around 90% of blind people live in developing countries. It is estimated that 75% of the cases of blindness in these countries could have been prevented but, in situations where people are poor and live in remote locations, both prevention and treatment efforts are extremely difficult. In times of war and civil conflict, the problems become even more severe. In these situations, it is very hard even to get an idea of the number of people who are blind. Surveys to find this out are important as a first step toward providing prevention and treatment services. Surveys play an essential part in international efforts to fight blindness.

**Why Was This Study Done?:**

Sudan is the largest country in Africa and one of the poorest in the world. Southern Sudan has spent most of the last five decades in a state of civil war and is a very remote region. The last information collected on the scale of the blindness problem was in the early 1980s. The researchers decided to conduct a survey in Mankien—a district of Sudan with a total population that is estimated to be around 50,000. Their aim was to estimate how many people were blind or had “low vision” and to find out the main causes of blindness. This would be useful in planning a blindness prevention programme for the district. It would also give some idea of the situation in the southern Sudan as a whole.

**What Did the Researchers Do and Find?:**

Working under very difficult conditions, the researchers selected villages to be visited at random. A house in each village visited was selected by spinning a pen in the middle of the village. The people in this house were examined and then other houses were chosen, also at random. In total, 2499 people were examined. Children under five years were not included in survey.

A very high rate of blindness was found—4%. This is more than twice the level that would be expected, given what is known about the prevalence of blindness in other parts of rural Africa. The two most common causes of blindness and low vision were cataract and trachoma, each accounting for over one-third of cases. Cataract is mainly a disease of older people; the lens of the eye becomes opaque. Trachoma is caused by an infection; it is the subject of another article by the same researchers in this issue of PLoS Medicine. Trachoma was responsible for a greater proportion of cases of blindness than has been found in studies in other parts of rural Africa.

**What Do These Findings Mean?:**

Based on the researchers' use of the random walk survey technique, the prevalence of blindness in this district and possibly the rest of southern Sudan appears to be extremely serious. The number of cases caused by trachoma is especially worrying. This information will help efforts to improve the situation. The implications of the study—and a discussion of the methods the researchers used—will be found in two “Perspective” articles in this issue of *PLoS Medicine* (by Buchan and by Kuper and Gilbert).

**Additional Information.:**

Please access these Web sites via the online version of this summary at http://dx.doi.org/10.1371/journal.pmed.0030477.

- General information about blindness is available on Wikipedia, an internet encyclopedia that anyone can edit
- Vision 2020 is a major international initiative to reduce blindness, in which many organizations collaborate
- The World Health Organization has a Web page on blindness
- Many charities provide help to blind people in developing countries, for example: Sight Savers, Lions Clubs International Foundation, Dark and Light Blind Care
- A profile of Sudan will be found on the website of the BBC

## Introduction

The World Health Organization (WHO) estimates that the number of people with visual impairment worldwide in 2002 was in excess of 161 million, of whom about 37 million were blind [1]. More than 90% of the world's visually impaired live in developing countries, the vast majority of them in rural areas of the least-developed countries [2]. In these settings, blindness is associated with considerable disability and excess mortality; resulting in huge economic and social consequences [3]. However, 75% of this visual impairment is estimated to be avoidable (preventable and curable) [4]. In 1999, the WHO Prevention of Blindness Program launched “VISION 2020: The Right to Sight Initiative” with the objective of assisting member states in eliminating avoidable blindness by the year 2020 [5,6]. The global target is to ultimately reduce blindness prevalence to less than 0.5% in all countries, or less than 1.0% in any community [7,8].

Baseline data on the prevalence and causes of blindness are important in planning for prevention-of-blindness strategies. Blindness is believed to be common in southern Sudan, although a national blindness survey has not been conducted. A blindness survey was conducted in East and West Equatoria provinces by Tizazu and Mburu in 1983; however, two interceding decades of civil war have rendered the data of limited use in guiding current prevention-of-blindness programs [9]. Reports from eye surgical camps and anecdotal data indicated that blindness and low vision were common in Mankien payam (district). This survey was conducted with the main objective of planning for the implementation of prevention-of-blindness programs in Mankien. The specific objectives were: (1) to estimate the prevalence of blindness and low vision; (2) to identify the main causes of blindness and low vision; and (3) to estimate targets for blindness prevention programs.

## Methods

### Study Site

**Figure 1:**
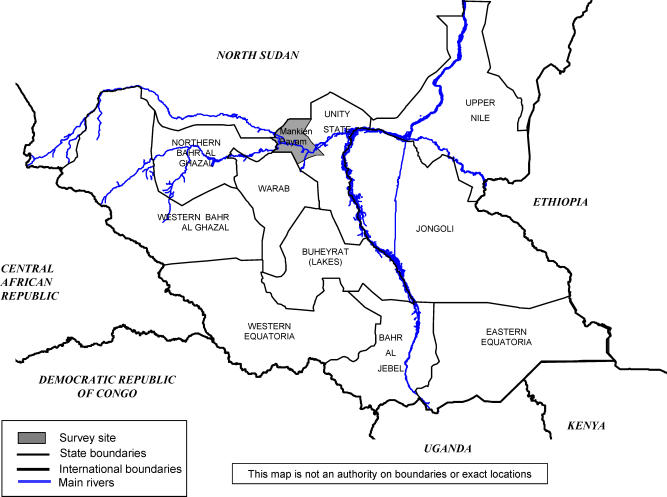
Map of Southern Sudan Showing the Study Site (Mankien Payam)

A population-based cross-sectional study was conducted in Mankien payam of southern Sudan in May 2005 (Figure 1, map). Mankien has an estimated population of 50,000 persons and is located in Unity state [10]. This area has been severely affected by the 21-y conflict in southern Sudan because it is located near the oil fields. Mankien comprises four bomas (subdistricts): Tam, Mandul, Kernyang, and Mankien. The main economic activities are cattle rearing and subsistence farming, and the Nuer are the predominant ethnic group. During the conflict, there were no eye care services available in the study area. However, in November 2004, an eye surgery camp was conducted by Christoffel-Blindenmission (CBM) during which training of integrated eye care workers (IECW) took place.

### Study Population

The study was conducted among persons aged 5 y and above. This target group was included because anecdotal data showed that blindness was common in both adults and children. The minimum age for visual acuity testing was predetermined to be 5 y.

### Sample Size and Sampling

There are no reliable data on blindness prevalence in southern Sudan on which to base sample-size calculations. We calculated our sample size assuming an expected blindness prevalence of 2.0% and worst acceptable prevalence of 1.0%. Using a 5% level of significance (two sided), 95% confidence limit, 90% power, and a design effect of 1.5, we estimated that at least 2,104 persons aged 5 y and above were to be examined [11–13]. A two-stage cluster random sampling design with probability proportional to size was used to select the sample. A cluster was defined as a village. The four bomas of Mankien payam have 73 villages. One boma (Mankien boma) comprising 12 villages was inaccessible at the time of the survey due to insecurity and was excluded from the sampling frame. In the first stage, 22 villages were randomly selected from the remaining 61 with probability proportional to the estimated population of the subdistrict. In the second stage, 25 households were selected from each sampled village using the random walk method, after selecting the first house by spinning a pen in the middle of the village [14]. All residents of selected households were enumerated and those present examined. An attempt was made to examine absentees by returning to households in which persons were absent on the day of the survey. Households in which all residents were not available were skipped. Due to logistical constraints, it was not possible to return to the village on a different day to follow up any absentees.

### Visual Acuity Testing and Basic Eye Examination

Integrated eye care workers (IECW) were retrained in visual acuity (VA) testing and basic eye examination by an experienced ophthalmic nurse (F. Ole-Sempele) over a period of 5 d. During the training, the minimum accepted interobserver agreement was set at 80% and reliability was assessed. Each trainee examiner was allowed to evaluate a set of 50 participants selected to represent varying VA (blindness, low vision, and normal vision), cataract, trichiasis, corneal opacity, and normal eye. Interobserver agreement was then calculated for each trainee examiner using the ophthalmic nurses' observation as the “gold standard.” Only trainees achieving an interobserver agreement of 80% and above were eligible to participate as examiners.

VA testing was conducted using the Snellen E chart at 6 m in adequate daylight, outdoors. In participants with VA less than 6/60, VA was evaluated with the Snellen chart at 3 m. Further VA assessment was done in participants with VA less than 3/60 by counting fingers, hand movement, and light perception, as appropriate. Basic eye examination was done in all persons after VA testing [2]. Using a torch and a 2.5× magnifying binocular loupe, each eye was examined separately for in-turned lashes (trichiasis), the cornea was then inspected for corneal opacities, and the lens examined for cataract. It was not possible to conduct detailed eye examination, intra-ocular pressure measurement, visual fields testing, and refraction, due to logistical and technical difficulties involved in conducting these examinations in the field set-up. Data were recorded on a customized form, and the cause of visual impairment determined by the examiner for all participants with a presenting VA of less than 6/18 for each eye separately. Participants who required surgical intervention or further assessment were referred to attend a surgical camp that was organized and conducted after the survey.

### Operational Definitions

The WHO categories of visual impairment were used to define vision status for study participants [15]. Blindness was defined as a presenting VA of less than 3/60 in the better eye. Low vision was defined as presenting VA of at least 3/60 but less than 6/18 in the better eye. Monocular visual impairment, which is not a WHO definition, was derived to represent participants who had normal or near-normal vision in the better eye (VA of at least 6/18) and visual impairment in the other eye (VA less than 6/18).

### Quality Control, Data Entry, and Analysis

After the survey, the ophthalmic nurse reviewed the forms and determined the principal disorder responsible for blindness or low vision for the participant, taking into account the main cause for each individual eye. In the instance when different causes had been identified for each eye separately in a given individual, the principal disorder was chosen to be the one that was most readily curable or, if not curable, most easily preventable. Data were double entered by different entry clerks and compared for consistency using EpiInfo version 3.3.2 (Centers for Disease Control and Prevention [http://www.cdc.gov/EpiInfo]). Statistical analysis was conducted using Stata version 8.2 (Stata Corporation [http://www.stata.com]). Pearson χ^2^ was used to assess the age and sex distribution of the sample population. Confidence intervals for the point estimates were derived using the Huber/White sandwich estimator of variance to adjust for clustering effects at the household level [16]. Sensitivity analysis of the prevalence estimates was undertaken by including all the enumerated persons in the denominator under the assumption that the absentees had no visual impairment; and the size of the change in prevalence estimates assessed. Inter-rater agreement of eye examination (VA and determination of cause) was assessed using the kappa *(κ)* statistic [17]. To derive population estimates of burden, prevalence estimates were adjusted for age and sex according to the sample population structure. The 95% confidence intervals (CIs) of the adjusted prevalence estimates were multiplied by the population estimates to derive the lower and upper bounds of those with blindness, low vision, and monocular visual impairment.

### Ethical Considerations

The Sudan People's Liberation Movement Secretariat of Health (SPLM/Health) approved the protocol, and clearance to conduct the surveys was obtained from the local authorities. Verbal consent to participate was sought from the head of the household, and from each individual, and the parents of small children in accordance with the declaration of Helsinki. Personal identifiers were removed from the dataset before analyses were undertaken.

## Results

### Sample Population

Twenty two villages (clusters) were sampled and 529 households visited. The mean household size was 7.5 (standard deviation [SD] = 2.5) with household size ranging from three to 16 persons. A total of 2,499 persons aged 5 y and above underwent VA testing and basic eye examination out of 2,954 persons enumerated, a response rate of 84.6%. Of the 2,499 persons included in the analysis, 1,038 (41.5%) were males and 1,461 (58.5%) were females (Table 1). The age and sex distribution among the 454 persons not examined was the same; however, there were more females than males aged 15 y and above among persons examined (Pearson χ^2^ = 41.1; *p*-value = 0.001). The age range was 5 y to 80 y, with a mean age of 23.9 y (SD = 16.6).

**Table 1:**
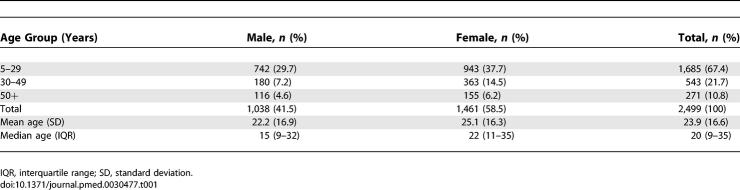
Demographic Characteristics of the Sample Population

### Prevalence of Blindness, Low Vision, and Monocular Visual Impairment

**Figure 2:**
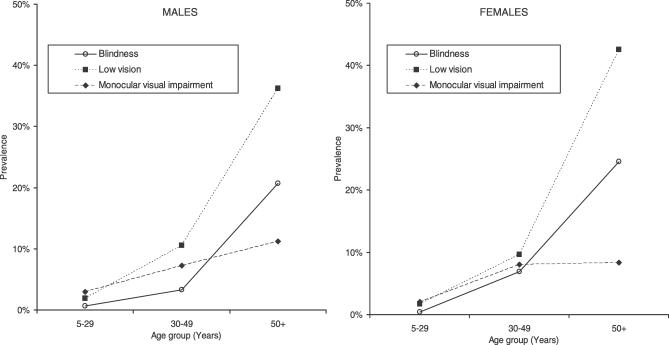
Age and Sex Distribution of Blindness, Low Vision, and Monocular Visual Impairment Left graph, males; right graph, females.

The age/sex-specific and overall prevalence of blindness, low vision, and monocular visual impairment found in this study are shown in Figure 2 and Table 2. Overall prevalence of blindness (VA of less than 3/60 in the better eye) was 4.1% (95% CI, 3.4–4.8). Prevalence of low vision (VA of at least 3/60 but less than 6/18 in the better eye) was 7.7% (95% CI, 6.7–8.7); whereas prevalence of monocular visual impairment (VA of at least 6/18 in the better eye and VA less than 6/18 in the other eye) was 4.4% (95% CI, 3.6–5.3). Prevalence of blindness and low vision increased in both males and females with age. There were no differences between the sexes in the odds of blindness (odds ratio [OR] = 1.38; 95% CI, 0.92–2.06), low vision (OR = 1.12; 95% CI, 0.82–1.53), and monocular visual impairment (OR = 0.90; 95% CI, 0.60–1.33). Sensitivity analysis of prevalence estimates by including 454 absentees in the denominator under the assumption that they had no visual impairment revealed prevalence of blindness = 3.5% (95% CI, 2.9–4.1), low vision = 6.5% (95% CI, 5.7–7.5), and monocular visual impairment = 3.7% (95% CI, 3.1–4.5). The size of change in prevalence was a 15% reduction based on prevalence in the 2,499 persons examined. Agreement of the examiners who participated in the survey against our standard (F. Ole-Sempele) ranged from 89% to 99%, and the overall kappa statistic was 0.61.

**Table 2:**
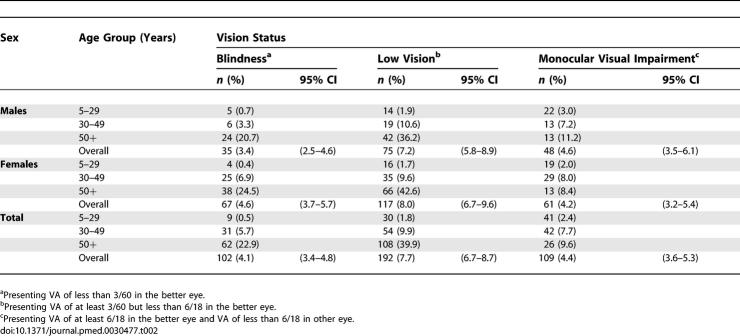
Prevalence of Blindness, Low Vision, and Monocular Visual Impairment by Age Group and Sex

### Causes of Blindness, Low Vision, and Monocular Visual Impairment

The main causes of visual impairment are summarized in Table 3. Cataract was the leading cause of blindness (41.2%), followed by trachoma (35.3%), nontrachomatous corneal opacity (18.6%), and other causes (4.9%). Low vision was caused mainly by trachoma (58.1%) and cataract (29.3%); whereas nontrachomatous corneal opacity and “other causes” accounted for 6.8% and 5.8% of low vision, respectively. Causes of monocular visual impairment were trachoma (37.6%), other causes (31.2%), cataract (22.0%), and nontrachomatous corneal opacity (8.3%). Trachoma was the leading cause of any form of visual impairment (46.8%), followed by cataract, other causes, and nontrachomatous corneal opacity at 30.3%, 12.7%, and 10.2%, respectively. Visual impairment due to cataract and trachoma increased markedly with increasing age. Other causes of visual impairment were more common in persons aged less than 30 y compared to those aged 30 y and above.

**Table 3:**
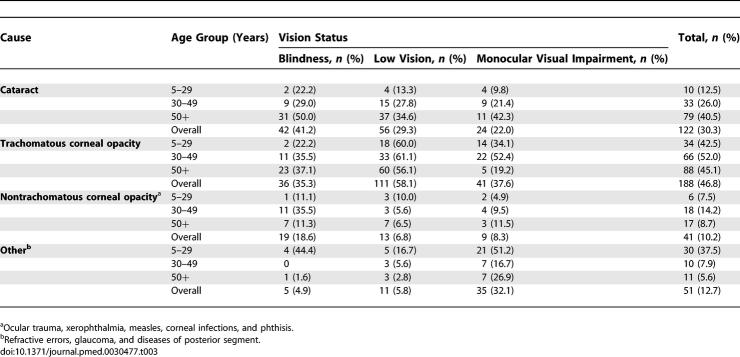
Main Causes of Blindness, Low Vision, and Monocular Visual Impairment by Age Group

### Burden Estimates of Visual Impairment

Burden estimates for all forms of visual impairment in Mankien payam are shown in Table 4. It was estimated that blindness affected 1,154 persons (lower and upper bounds = 782–1,799). Low vision affected 2,291 persons (lower and upper bounds = 1,820–2,898); whereas 1,556 persons (lower and upper bounds = 1,145–2,152) had monocular visual impairment. Therefore, up to 6,849 persons (13.7% of target population) in Mankien payam were estimated to have some form of visual impairment.

**Table 4:**
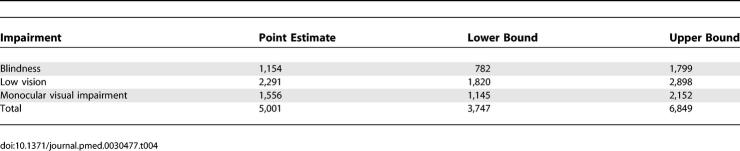
Estimates of Persons with Visual Impairment in Mankien Population (*n* = 50,000)

## Discussion

This population-based survey provides some of the first contemporary survey data on the prevalence of blindness in southern Sudan, and is consistent with the initial reports that blindness is a severe public health problem in Mankien payam. The study provides data for priority setting and planning for prevention of blindness activities in Mankien. The prevalence of blindness in persons aged 5 y and above was 4.1% whereas low vision prevalence was 7.7%. The prevalence was comparable between males and females. The main causes of blindness were considered to be cataract (41.2%) and trachoma (35.3%). Three fifths of low vision was caused by trachoma and a third by cataract. For planning purposes, it is estimated that up to 6,849 persons had some form of visual impairment in the study population. These data will be used for estimating intervention goals for primary eye care services, eye surgery, trachoma control, and rehabilitation of the blind.

Our study was conducted in a logistically challenging postconflict setting and has several limitations associated with the difficulty of doing field studies in this environment. Twelve villages had to be excluded from the sampling frame because they were considered insecure, and clearance to enter them was denied. Although this is a potential source of bias, we do not expect this to have affected the validity of this survey given the homogenous nature of the payam and the potential risk factors that predispose these communities to blindness. We used the random walk method to select households because it was considered the most practical given the terrain, absence of maps, and difficulty of using geographic positioning systems (GPS). The random walk method, although acceptable for other purposes, is not ideal where the outcome being assessed is one that is obvious to those involved in guiding the survey teams. Bias could have been introduced because the village guides may have been more likely to direct the survey teams to households where they knew there were persons with visual impairment. A response rate of 84.6% was achieved, which was considered adequate to meet the study objectives. It was not possible to go back to the villages on a different day to follow up absentees in participating households and households in which all members were not present on the day of the survey. Therefore, not including absentees in the sample may have resulted in selection bias since absenteeism was possibly associated with normal vision or less-severe visual impairment. Attempts were not made to assess visual impairment in absentees: reports by households' members on vision status of those absent were considered unreliable and were likely to be biased. It is possible that the high prevalence of blindness observed in Mankien could have been an overestimation resulting from selection bias due to use of the random walk method, exclusion of absentees, and exclusion of households in which nobody was found for enumeration. Sensitivity analysis adjusting prevalence estimates by including absent persons in the denominator on the assumption that they did not have visual impairment showed an upper bound for the degree of overestimation of 15%.

Assessment of vision status and causes of vision loss were based on basic examination of the eye because it was not possible to conduct refraction and detailed eye examination, due to logistical constraints. It was also not logistically possible for the ophthalmic nurse to re-examine VA and causes of visual impairment in all participants with visual impairment; therefore a reliability study was conducted prior to the survey. Six out of eight examiners were integrated eye care workers (IECW) who had good interobserver reliability compared to our standard, and there was no evidence of systematic examiner bias. We have reported vision status based on presenting VA and not best-corrected VA, which is consistent with a recent WHO recommendation of measuring presenting VA in blindness surveys [1]. Defining vision status based on presenting VA is not a potential source of bias because our results include vision-disabling refractive errors. In most blindness surveys of sub-Saharan Africa, natural refractive error has not been shown to be a considerable cause of blindness; nevertheless it is a major cause of low vision [18]. Our data on causes of vision loss do not allow for detailed differential diagnosis in persons with visual impairment. Therefore the causes of visual impairment may be biased towards the most preventable causes due to the study design. Nonetheless, these data underscore the severity of visual impairment and highlight the main causes of avoidable blindness in this population, thus allowing for planning of interventions within the VISION 2020 objectives.

The WHO considers blindness to be a public health problem when the prevalence of blindness in the general population exceeds 1.0% [7,8]. The prevalence of blindness revealed in Mankien payam greatly exceeds this WHO parameter, and is consistent with that reported in studies conducted in rural settings of northern Sudan: Al-Ginena province (3.2%) and River Nile State (2.74%) [19]. A study conducted in East and West Equatoria provinces of southern Sudan in 1983 reported a blindness prevalence of 6.4%; however, the sampling frame for this study was not well defined and the definition of blindness—VA less than 6/60 in the better eye—was not consistent with the WHO definition of blindness: VA less than 3/60 in the better eye [9]. Blindness prevalence in Mankien also exceeds that reported in other settings in sub-Saharan Africa: the Gambia (0.7%) [20], Nigeria (0.3%–0.9%) [21,22], Malawi (1.3%) [23], Tanzania (1.3%) [24], Kenya (0.7%) [25], and Ethiopia (0.9%–1.9%) [26–28]. Cataract was considered the commonest cause of blindness in Mankien (41.2%). This is consistent with findings from other countries in Africa where cataract has been found to be the leading cause of blindness: Kenya (38%) [25], Nigeria (48%) [22], Malawi (40%) [23], and the Gambia (45%) [20]. However, trachoma was almost as common a cause of blindness as cataract (35.3%) and was the leading cause of all forms of visual impairment (46.8%). The proportion of blindness due to trachoma by far exceeds what has been observed in other countries where trachoma is endemic: Mali (12.1%) [29], Kenya(18.7%) [25], Ethiopia (20.6%) [27], and Tanzania (26%) [24]. In Mankien, trachomatous trichiasis (TT) has been documented in children as young as 4 y, overall (all ages) TT prevalence was 9.6%, and prevalence of bilateral trachomatous corneal opacity was 3.1% [30]. Nontrachomatous corneal opacity accounted for a fifth of blindness and a tenth of all forms of visual impairment. Although the etiology of such corneal scarring in adults is often difficult to ascertain, the histories obtained suggested that ocular trauma, vitamin A deficiency, measles, and corneal infections were the most likely causes.

We observed a lower than expected prevalence of monocular visual impairment and a low ratio of blindness to low vision. This atypical picture may partly be explained by severe and early onset of blinding trachoma, accumulation of blindness in the absence of eye care services, as well as over sampling of blind people. Mankien was not randomly selected to represent southern Sudan, but was surveyed on the basis of need in response to a report from the field. Nonetheless, our data probably underscore a severe problem in possibly the entire region because the conflict affected all areas, and basic eye care service has been all but absent for over 20 y. Therefore, further surveys are essential to confirm these findings and to estimate the prevalence, determine causes, and investigate the pattern of blindness and low vision in the entire region of southern Sudan.

The study area has blindness of severe public health magnitude with over 4-fold prevalence compared to WHO parameters. The high prevalence of low vision and monocular visual impairment predicts a greater prevalence of blindness in the study area in the future. There is urgent need to target blindness prevention interventions in this population. This will involve setting up cataract surgical services, trachoma control programs, vitamin A supplementation, and optometry services. There is also need to put in place interventions to rehabilitate persons whose blindness is not reversible, especially those blinded by trachoma.

## Abbreviations

CI: confidence interval
IECW: integrated eye care workers
WHO: World Health Organization
VA: visual acuity
