# Correction: Prevalence and Causes of Blindness and Low Vision in Southern Sudan

**DOI:** 10.1371/journal.pmed.0040227

**Published:** 2007-06-26

**Authors:** Jeremiah Ngondi, Francis Ole-Sempele, Alice Onsarigo, Ibrahim Matende, Samson Baba, Mark Reacher, Fiona Matthews, Carol Brayne, Paul M Emerson

In *PLoS Medicine*, volume 3, issue 12, doi:10.1371/journal.pmed.0030477:

In the Methods and Findings section of the Abstract, the figures 18/60 in the following sentence are incorrect and should read 6/18 to be consistent with what is in the main text of the article:

“Prevalence of blindness (presenting VA of less than 3/60 in the better eye) was 4.1% (95% confidence interval [CI], 3.4–4.8); prevalence of low vision (presenting VA of at least 3/60 but less than 18/60 in the better eye) was 7.7% (95% CI, 6.7–8.7); whereas prevalence of monocular visual impairment (presenting VA of at least 18/60 in better eye and VA of less than 18/60 in other eye) was 4.4% (95% CI, 3.6–5.3).”

Please note that this error does not change the data or the conclusions in the paper. It is an error in the abstract only.

